# Circulating microRNAs correlate with structural and functional MRI parameters in patients with multiple sclerosis

**DOI:** 10.3389/fnmol.2023.1173212

**Published:** 2023-10-10

**Authors:** Lili Geiger, Gergely Orsi, Tamás Cseh, Katalin Gombos, Zsolt Illés, Boldizsár Czéh

**Affiliations:** ^1^Department of Laboratory Medicine, Medical School, University of Pécs, Pécs, Hungary; ^2^Szentágothai Research Centre, University of Pécs, Pécs, Hungary; ^3^HUN-REN-PTE Clinical Neuroscience MR Research Group, Eötvös Loránd Research Network, Pécs, Hungary; ^4^Department of Neurology, Medical School, University of Pécs, Pécs, Hungary; ^5^Pécs Diagnostic Centre, Pécs, Hungary; ^6^Department of Neurology, Odense University Hospital, Odense, Denmark; ^7^Institute of Clinical Research, University of Southern Denmark, Odense, Denmark

**Keywords:** default mode network, droplet digital PCR, magnetic resonance imaging, miR-92a, mIR-142, miR-143, miR-486, resting-state functional MRI

## Abstract

**Introduction:**

Circulating microRNAs are promising biomarkers for multiple sclerosis (MS). Our aim was to correlate serum microRNA levels with various magnetic resonance imaging (MRI) parameters.

**Methods:**

We recruited 50 MS patients and measured cervical spine and cerebral white matter lesions together with regional brain volumes. Microstructural changes in the white matter were investigated with diffusion tensor imaging. Magnetic resonance spectroscopy was performed to measure cerebral metabolites. Functional connectivity within the default mode network was examined with resting-state functional MRI. On the day of the MRI measurements, we collected serum samples and carried out quantitative analysis of ten pre-selected microRNAs using droplet digital PCR.

**Results:**

Serum level of miR-143.3p could differentiate between MS subtypes and had lower levels in progressive MS types. We found significant associations between microRNA levels and MRI measures: (1) higher miR-92a.3p and miR-486.5p levels were associated with greater total white matter lesion volumes within the cervical spine, (2) decreased miR-142.5p levels was associated with reduced total creatinine concentration and (3) miR-92a.3p, miR-142.5p and miR-486.5p levels were associated with functional connectivity strengths between specific nodes of the default mode network. Specifically, we found a negative association between miR-92a.3p and miR-486.5p levels and connectivity strength between the lateral temporal cortex and posterior inferior parietal lobule, and a positive association between miR-142.5p level and connectivity strength between the retrosplenial cortex and temporal pole. However, miRNA levels were not associated with regional brain volumes.

**Conclusion:**

We provide here further evidence that circulating microRNAs may show correlation with both structural and functional neuroimaging outcomes in patients with MS.

## 1. Introduction

Multiple sclerosis (MS) is an inflammatory neurodegenerative disorder affecting individuals with increasing incidence and prevalence (Dobson and Giovannoni, 2019). Recent epidemiological studies estimate around 2.8 million cases of MS worldwide and report on steady increase of prevalence, MS related deaths, and disability-adjusted life years (DALYs) in every world region, especially in high-income countries (GBD 2016 Multiple Sclerosis Collaborators, 2019; Walton et al., 2020; Qian et al., 2023). Due to the scarcity of biomarkers with the disease course, there is a pressing need to develop novel biomarkers to assess disease prognosis and predict treatment response.

Magnetic resonance imaging (MRI) is the best diagnostic tool to detect and monitor the number, size, and location of focal white matter lesions in central nervous system (CNS), but additional MRI-based methods are increasingly utilized to examine further abnormalities specific to MS (Filippi, 2015; Filippi et al., 2019). Volumetric MRI analysis can reveal global or regional tissue atrophy (Anderson et al., 2006; Orsi et al., 2021b), and diffusion tensor imaging can document widespread microstructural alterations in the normal-appearing white matter (Werring et al., 1999; Huang et al., 2018; Orsi et al., 2021a). Proton magnetic resonance spectroscopy (^1^H-MRS) reveals metabolic changes in both white and gray matter structures and may identify a metabolic signature specific to MS (Huang et al., 2018; Swanberg et al., 2019). Resting-state functional MRI studies report on large-scale and within network alterations of functional connectivity in MS (Jandric et al., 2022).

MicroRNAs (miRNAs) are short (18–23 nucleotides), non-coding single-stranded RNA molecules that can regulate gene expression at the post-transcriptional level. Current interest focus on the miRNA-expression profiles since these may serve as novel diagnostic and prognostic biomarkers for multiple human diseases. A rapidly growing number of studies report on dysregulated miRNAs in various biofluids of MS patients which seem to have the potential to distinguish the disease phenotypes from each other (Gandhi et al., 2013; Martinez and Peplow, 2020; Pietrasik et al., 2021). However, there is only a single study which focused exclusively on comparing circulating miRNAs levels to MRI outcomes in MS patients. That cross-sectional American study recruited 120 MS patients with three main disease categories (95 relapsing–remitting MS, 17 secondary-progressive MS, and 8 primary-progressive MS) and compared brain and spinal cord lesion volumes and tissue atrophy of these structures with serum miRNA levels (Regev et al., 2017). The authors found that numerous miRNAs were associated with MRI outcomes, but none of these associations remained significant when they corrected for multiple comparisons therefore, they concluded that further studies are needed to validate their findings. A few, more recent studies also reported on associations between circulating miRNA levels and volumes of specific brain structures (Liguori et al., 2019; Dominguez-Mozo et al., 2022).

The primary aim of our study was to extend the earlier data and to find correlations between circulating microRNA levels and results of additional MRI measurements. Therefore, we performed a series of MRI measurements to correlate circulating miRNA levels with brain and spinal cord MRI abnormalities in MS patients. We quantified cervical spine and cerebral white matter lesions and measured 40 regional brain volumes. Microstructural alterations in the white matter were examined with diffusion tensor imaging using the tract-based spatial statistics approach and single-voxel MR spectroscopy was performed to measure cerebral metabolites. Functional connectivity within the default mode network was examined using resting-state functional MRI. Based on the earlier findings of Regev and co-workers, we selected ten miRNAs (Table 1) focusing on the ones that had the most robust associations with CNS lesions and atrophy in the Regev et al. study (2017). Regev and co-workers investigated two independent cohorts involving 41 and 79 patients and they found eight miRNAs which showed significant correlations with the MRI findings in both cohorts: miR-92a.3p, miR-142.5p, miR-143.3p, miR-181c.3p, hsa-miR-181c.5p, miR-375, miR-486.5p, and miR − 629.5p. Clearly, these eight miRNAs showed the strongest correlation with lesion volume and brain atrophy. We selected these eight miRNAs and we included two more miRNAs: miR-21.3p and miR-146a, since these also had significant correlations with the MRI measures in the smaller cohort (Cohort 1) of the same Regev study. Another study also reported upregulation of miR-21 and miR-146a in the cerebrospinal fluid (CSF) of MS patients with active lesions (Muñoz-San Martín et al., 2019). Furthermore, miR-146a seems to play a pivotal role in regulating the homeostasis of the neuroimmune system and shows altered expression and function in multiple neurological diseases (Mortazavi-Jahromi et al., 2020; Aslani et al., 2021). Notably, all these ten miRNAs have already been identified by earlier studies as potentially significant contributors to the pathogenesis of SM, or as potentially useful biomarkers for the diagnosis (Table 1). Our hypothesis was that these ten circulating miRNA levels would correlate with structural MRI data (lesion volume, brain atrophy and white matter microstructure) and possibly also with the functional MRI findings (functional connectivity strengths and brain metabolite concentrations).

**Table 1:** The ten pre-selected miRNAs investigated in this study.

miRNA	Main findings of the Regev et al. (2017) study*	Known functional role in the pathogenesis or diagnosis of MS	Number of patients with detectable miRNA expression levels in our study	Main findings of our study
hsa-miR-21.3p	Protective correlation with brain atrophy in cohort 1.	Increased level in cell-free cerebrospinal fluid (CSF) of MS patients with active lesions (Muñoz-San Martín et al., 2019). MiR-21 was upregulated in active MS lesions (Junker et al., 2009).	14	Excluded **
hsa-miR-92a.3p	Positive (pathogenic) correlation with brain lesion (T1:T2 ratio) in both cohorts.	Different expression of miR-92a.1 was detected in RRMS versus healthy controls and SPMS patients (Gandhi et al., 2013). MiR-92a.1 showed an association with EDSS scores and disease duration (Gandhi et al., 2013). MiR-92a was elevated in CD4+ T cells from patients with MS and it has been suggested to play a major disease-promoting role in CNS autoimmunity (Fujiwara et al., 2022).	26	Positive association with total white-matter lesion volume within the cervical spine. Negative association with functional connectivity strength between the lateral temporal cortex and posterior inferior parietal lobule.
hsa-miR-142.5p	Negative (protective) correlation with brain lesion (T1:T2 ratio) in both cohorts.	Upregulated in active MS lesions (Junker et al., 2009). Serum levels were upregulated in RRMS samples compared with other neurological disorders (Muñoz-San Martín et al., 2022). Lower CSF levels were associated with higher number of T2 lesions and increased CSF levels correlated with higher number of gadolinium+ active lesions (Muñoz-San Martín et al., 2022). Increased expression in the frontal white matter from MS (Talebi et al., 2017). Increased levels of miR-142-3p (an isoform of miR-142.5p) in the CSF of patients with active MS (Mandolesi et al., 2017). Significant difference between patients with SPMS and healthy individuals (Regev et al., 2018). Correlates with EDSS scores (Regev et al., 2018).	30	Positive association with total creatinine concentrations in the brain. Positive association with functional connectivity strength between the retrosplenial cortex and temporal pole. Negative correlation with disease duration.
hsa-miR-143.3p	The highest negative (protective) correlation with brain lesion (T1:T2 ratio) in both cohorts.	CSF levels were downregulated in PPMS samples compared with other neurological disorders (Muñoz-San Martín et al., 2022).	46	Lower expression levels in SPMS patients compared to RRMS and PPMS patients. Positive correlation with EDSS scores.
hsa-miR-146a	Protective correlation with brain atrophy (global cerebral gray matter fraction) in cohort 1.	Upregulated in active MS lesions (Junker et al., 2009). Increased level in cell-free CSF of MS patients with active lesions (Muñoz-San Martín et al., 2019).	3	Excluded **
hsa-miR-181c.3p	Negative (protective) correlation with brain lesion (T1:T2 ratio) in both cohorts.	MiR-181c was downregulated in active MS lesions (Junker et al., 2009). MiR-181c was dysregulated in patients with MS as compared with patients with other neurologic diseases and it differentiated RRMS from SPMS with high specificity and sensitivity (Haghikia et al., 2012). High CSF levels of miRNA-181c were associated with conversion from clinically isolated syndrome to RRMS (Ahlbrecht et al., 2016).	5	Excluded **
hsa-miR-181c.5p	Negative (protective) correlation with brain lesion (T1:T2 ratio) in both cohorts.	Reduced levels were measured in peripheral blood mononuclear cells (Martinelli-Boneschi et al., 2012). Downregulated in active MS lesions (Junker et al., 2009).	2	Excluded **
hsa-miR-375	Negative (pathogenic) correlation with brain atrophy in both cohorts.	Downregulated in the serum of PPMS patients compared to healthy controls (Vistbakka et al., 2017).	3	Excluded **
hsa-miR-486.5p	Positive (pathogenic) correlation with brain lesion (T1:T2 ratio) in both cohorts.	Correlates with EDSS scores, upregulated in MS compared to healthy controls and to other neurologic diseases, as well as compared to other inflammatory diseases (Regev et al., 2016). Significant difference between patients with MS and healthy individuals (Regev et al., 2018).	41	Positive association with total white-matter lesion volume within the cervical spine. Negative association with functional connectivity strength between the lateral temporal cortex and posterior inferior parietal lobule.
hsa-miR-629.5p	Negative (pathogenic) correlation with brain atrophy in both cohorts.	Significantly deregulated in patients with clinically isolated syndrome, or with RRMS (Keller et al., 2014). MiR-629 was upregulated in inactive MS lesions (Junker et al., 2009).	7	Excluded **

*The Regev et al. (2017) study involved two independent cohorts with 41 and 79 patients and with three main disease categories (relapsing–remitting MS, secondary-progressive MS, and primary-progressive MS) and compared brain and spinal cord lesion volumes and tissue atrophy of these structures with serum miRNA levels. To assess the destructive potential of the lesions, they calculated an index, i.e., the intra-patient ratio of T1LV to T2LV (T1:T2). **These six miRNAs were excluded from the statistical comparisons with the MRI measurements because of their low detectability in the serum. CSF, cerebrospinal fluid; EDSS, expanded disability status scale; PPMS, primary-progressive multiple sclerosis; RRMS, relapsing–remitting multiple sclerosis; SPMS, secondary-progressive multiple sclerosis.

## 2. Materials and methods

### 2.1. Participants

The present study was a cross-sectional, single-center study, carried out at the Medical School, University of Pécs, Hungary. Patients with MS (*n* = 50) were recruited consecutively, using the total enumerative sampling technique between 2018 and 2019. We included all suitable patients from the available clinical pool of the Department of Neurology, Medical School, University of Pécs. The diagnosis was made by an experienced neurologist T.C. according to the 2017 modified McDonald diagnostic criteria (Thompson et al., 2018). Demographic data of the patients are presented in Table 2. The age of the patients ranged between 20 and 68, with a mean age of 44. The sex ratio was the following: 66% (*n* = 33) of the patients were women and 34% were men (*n* = 17). Results from this cohort have been published earlier (Orsi et al., 2021a,b).

**Table 2:** Clinical characteristics of the MS patients.

Characteristics	Number of patients, mean ± SD or median (IQR)
Demographics	
Number of patients	50
Disease duration (years)	13 (8–15)
Age at onset (years)	31 ± 10
Sex	
Male	17 (4%)
Female	33 (66%)
Disease type (number of patients)	
PPMS	7 (14%)
SPMS	14 (28%)
RRMS	29 (58%)
EDSS at the time of serum collection	2 (1–4.5)
DMT at the time of MRI	
None	16 (32%)
Interferon-beta	12 (24%)
Fingolimod	5 (10%)
Dimethyl fumarate	3 (6%)
Teriflunomide	4 (8%)
Glatiramer acetate	8 (16%)
Other (alemtuzumab, ocrelizumab, azathioprine)	2 (4%)
Mental health	
Beck depression Interventory-II	6 (4–13)
State–Trait Anxiety Inventory (S)	38 (31–45)
State–Trait Anxiety Inventory (T)	39 (33–44)

Data with normal distribution are reported as mean ± SD. Data without normal distribution are reported as median (25–75% interquartile range). DMT, disease modifying therapy; EDSS, Expanded Disability Status Scale; IQR, interquartile range; PPMS, primary-progressive multiple sclerosis; RRMS, relapsing–remitting multiple sclerosis; SD, standard deviation; SPMS, secondary-progressive multiple sclerosis.

The local Research Ethics Committee of the University of Pécs approved the study design and protocol (Ethical Approval Nr.: 2018/7503-PTE and 2018/7068-PTE). All patients signed written informed consent.

### 2.2. Blood sampling and general molecular laboratory methods

Venous blood samples were collected on the same day of the MRI measurements from each patient. Shortly after collection the samples were centrifuged for 10 min with 3,000 g and then, serum samples were stored at −80°C before further processing. All molecular methods were carried out by a single experimenter L.G., in sterile, RNase free laboratory environment to avoid RNA degradation or sample contamination.

### 2.3. RNA isolation and reverse transcription

Cell free total RNA was isolated from 500 μl of serum sample using the miRNeasy serum/plasma kit (Qiagen, Hilden, Germany). Isolated RNA quality was verified by using Thermo Scientific NanoDropTM 2000 (Thermo Fisher Scientific, Waltham, USA). Five ng total RNA from each sample was reverse transcribed with miRCury LNA Universal RT microRNA PCR Kit (Qiagen, Hilden, Germany), applying random hexamer priming.

### 2.4. Droplet digital PCR (ddPCR) analysis

For quantitative PCR analysis we purchased Qiagen miRCury LNA miRNA PCR assays (Qiagen, Hilden, Germany). For greater sensitivity, specific LNA-enhanced primers were used according to target miRNAs. We investigated the following specific miRNAs: hsa-miR-21.3p, hsa-miR-92a.3p, hsa-miR-142.5p, hsa-miR-143.3p, hsa-miR-146a, hsa-miR-181c.3p, hsa-miR-181c.5p, hsa-miR-375, hsa-miR-486.5p, and hsa-miR-629.5p. PCR master mixes were prepared containing 1 μl of miRCury LNA miRNA assay containing the target-miRNA specific forward and reverse primer pair, 12 μl of QX200 ddPCR EvaGreen Supermix (Bio-Rad) and 9 μl PCR grade water and 2 μl (~100 pg) of cDNA sample. NTC (no template control) and NPC (no primer control) were used as negative control, water was added to master mix instead of the template cDNA and primer.

Droplets were generated from each master mix sample in the automated droplet generator unit of QX200 automated ddPCR system using Droplet Generation Oil for EvaGreen (Bio-Rad, CA, USA). PCR amplification was carried out in the QX200 Thermo cycler with the following thermic conditions: enzyme activation 1 cycle (95°C for 5 min); amplification 40 cycles (96°C for 30s and 58–60°C for 1 min); signal stabilization 1 cycle (4°C for 5 min and 90°C for 5 min) and hold on 4°C. After the PCR reaction droplets were analyzed in the QX200 Droplet Reader. Fluorescence data were converted into concentrations according to the Poisson distribution statistical analysis used by the QuantaSoft® analysis Pro software version 1.0.596 (Bio-Rad, CA, USA).

### 2.5. Magnetic resonance imaging

A whole-body 3Tesla MRI scanner was used during the study (MAGNETOM Prisma^Fit^, Siemens Healthineers, Erlangen, Germany). The following sequences were included in the prospective MRI study: 3D T1 magnetization-prepared rapid acquisition with gradient echo (MPRAGE), 3D fluid-attenuated inversion recovery (FLAIR), diffusion tensor imaging (DTI), single voxel Point RESolved Spectroscopy (MRS), phase-sensitive inversion recovery (PSIR) imaging of the cervical spine region and resting-state functional MRI (rs-fMRI) with field mapping to reduce image distortions due to B0 inhomogeneities. Sequence parameters were as follows: MPRAGE (TR/TI/TE = 2530/1100/3.37 ms; Flip Angle = 7°; 176 sagittal slices; slice thickness = 1 mm; FOV = 256×256 mm^2^; matrix size = 256×256; receiver bandwidth = 200 Hz/pixel), FLAIR (TR/TI/TE = 5000/1800/387 ms; variable flip angle; 192 sagittal slices; slice thickness = 0.9 mm; FOV = 230×230 mm^2^; matrix size = 256×256; receiver bandwidth = 751 Hz/pixel), DTI (TR/TE = 7700/68 ms; 70 axial slices; slice thickness = 2 mm; FOV = 256×256 mm^2^; matrix size = 128×128; receiver bandwidth = 1,562 Hz/pixel; 30 diffusion directions with homogeneous distribution on a half sphere with a *b*-value of 1,000 s/mm^2^ and 6 volumes were collected with no diffusion gradients applied; all images were collected twice with opposite phase-encoding directions), MRS (TR/TE = 3000/30 ms; 80 averages; receiver bandwidth = 1,200 Hz; delta frequency = −2.7 ppm; vector size = 1,024; voxel size = 15 mm isotropic; voxel position: normal appearing white matter of the centrum semiovale) (separate water signal was also recorded for quantitative metabolite concentration assessment), PSIR (TR/TI/TE = 2400/400/9.4 ms; Flip Angle = 160°; 15 sagittal slices; slice thickness = 3 mm; FOV = 220×220 mm^2^; matrix size = 320×320; receiver bandwidth = 220 Hz/pixel), rs-fMRI (TR/TE = 2000/30 ms; 36 axial slices; Flip Angle = 76°; slice thickness = 3 mm; number of volumes = 300; FOV = 210×210 mm^2^; matrix size = 70×70; receiver bandwidth = 2040 Hz/pixel).

### 2.6. Tract-based spatial statistics

Tract-based spatial statistics (TBSS) v1.2 was used for the voxel-wise analyses (Smith et al., 2006). Image quality of the diffusion data was visually assessed for each patient. Subjects with excessive within volume movement artefacts or any other serious image quality degradation in the raw or calculated diffusion data were excluded from further analyses. Susceptibility-induced off-resonance field and undistorted b = 0 s/mm^2^ (b0) images were calculated by TOPUP (part of FSL 5.0.11) (Andersson et al., 2003; Smith et al., 2004). After brain-extraction using FSL-BET (Smith, 2002), the CUDA implementation of FSL eddy (eddy_cuda7.5) was used to correct for susceptibility-induced distortions, eddy currents and subject motion, and to perform outlier detection and replacement for slices with average intensity ≥3 standard deviations lower than expected (Andersson et al., 2016; Andersson and Sotiropoulos, 2016). Positive outliers were not assessed. The average percentage of outlier slices was 0.49% of all slices (range: 0–1.4%). Finally, FSL’s DTIFit was used to calculate fractional anisotropy (FA), mean diffusivity (MD) and radial diffusivity (RD) images from the pre-processed DTI data (Smith et al., 2004). After the pre-processing, the “most typical” FA image was identified among the patients based on the criteria that the smallest amount of average warping was necessary to align all other FA images to it (i.e., target image). Each subject’s FA image was transformed into the 1 mm isotropic resolution MNI152 space by combining the non-linear transformation to the target image with the affine transformation from the target to MNI152 space (Jenkinson et al., 2002; Andersson et al., 2007). Then, the mean FA image was created and used to generate a mean FA skeleton representing the central voxels of all tracts. This mean skeleton was thresholded at FA > 0.2. FA, MD, AD, and RD maps were then projected onto this skeleton by filling the skeleton with the values from the nearest relevant tract center. A lesion map was also created from all patients to exclude areas with visible white matter (WM) lesions (no threshold were applied here). Voxel-wise general linear models were applied on the resulting skeletons using permutation-based non-parametric testing (10,000 permutations) with miRNA amount as variable of interest and gender, age, and current therapy as covariates of no interest (Winkler et al., 2014). Results were considered significant for *p* ≤ 0.05, fully corrected for multiple comparisons (−-T2 option in randomize).

### 2.7. Segmentation of cervical spine and cerebral white matter lesions

Cervical spine lesions were manually segmented on sagittal PSIR images using 3DSlicer (4.10.2 r28257). Level tracing option was used within the editor and the resulting labels were manually corrected. Label statistics were used to export the number of lesions and the volume of each lesion for statistical evaluation. Cerebral white matter lesions were segmented on 3D FLAIR images using the lesion prediction algorithm (Schmidt, 2017) as implemented in the freely available LST toolbox version 3.0.0 (Lesion Segmentation Toolbox^1^) for SPM (SPM12).

### 2.8. Automated MRI volumetry

FreeSurfer v6.0 was used for the automated segmentation of high resolution T1-weighted MPRAGE images. Details of the procedures are described in previous publications (Fischl et al., 2002, 2004). Each dataset was checked within the processing stream to verify the following stages: Talairach transform, skull strip, white matter-and pial surface segmentation, as described in Freesurfer’s Recommended Reconstruction Workflow. The volumetric results from aseg.mgz were fed into statistical analysis.

### 2.9. Resting-state-fMRI evaluation

Resting-state-fMRI data were pre-processed using DPARSF 5.0, part of DPABI (V4.3_200401^2^) (Yan et al., 2016) and SPM 12. Functional connectivity was automatically calculated between the 11 regions of interest (ROIs) of the Default Mode network (DMN) based on the Andrews-Hannah DMN atlas (Andrews-Hanna et al., 2010) along with the amplitude of low-frequency fluctuation (ALFF) maps for all subjects. The z-standardized functional connectivity values between the predefined ROIs were exported for further statistical analyses. Within the DMN, fifty-five connections were examined altogether ( $\frac{n*\left(n-1\right)}{2}$ ). See Table 3 for the names, abbreviations, and MNI coordinates of DMN ROIs. The first 10 time points of each subject were excluded to achieve signal equilibrium. The remaining data were slice-time corrected. Motion correction was performed by using the Friston-24 model for individual-level correction, and any subject with mean framewise displacement (FD_Jenkinson_) > 0.2 was excluded. Both T1 and functional datasets were reoriented, T1 datasets were brain-extracted using BET (Smith, 2002) and T1 images were co-registered to the functional datasets. The resulting 4D functional volumes were then spatially normalized to Montreal Neurological Institute (MNI) space, using Diffeomorphic Anatomical Registration Through Exponentiated Lie algebra (DARTEL) (Ashburner, 2007). After the normalization, linear trends were removed accounting for scanner drift and multiple linear regression was performed on potential nuisance variables including head motion parameters, cerebrospinal fluid, and white matter signals. Due to controversies regarding the removal of global signal (Scholvinck et al., 2010; Chai et al., 2012), the global signal was not included in the multiple linear regression model. Finally, spatial smoothing was employed with FWHM of 4 × 4 × 4 mm. Functional connectivity was automatically calculated between the 11 ROIs of the DMN. ROIs were formed with a radius of 4 mm centered around the coordinates listed in Table 3. ALFF was also calculated using a temporal band-pass filtering (0.01–0.1 Hz). ALFF maps were fed into randomize (Winkler et al., 2014) creating voxel-wise general linear models with miRNA amount as variable of interest and gender, age, and current therapy as covariates of no interest. Results were considered significant for *p* ≤ 0.05, fully corrected for multiple comparisons (−-T option in randomize).

**Table 3:** Region of interests of the default mode network.

Name	Abbreviation	MNI *x*	MNI *y*	MNI *z*
Anterior medial prefrontal cortex	aMPFC	–6	52	–2
Dorsomedial prefrontal cortex	dMPFC	0	52	26
Hippocampal formation	HF	−22	−20	−26
Lateral temporal cortex	LTC	−60	−24	−18
Parahippocampal cortex	PHC	−28	−40	−12
Posterior cingulate cortex	PCC	−8	−56	26
Posterior inferior parietal lobule	pIPL	−44	−74	32
Retrosplenial cortex	Rsp	−14	−52	8
Temporal parietal junction	TPJ	−54	−54	28
Temporal pole	TempP	−50	14	−40
Ventromedial prefrontal cortex	vMPFC	0	26	−18

Names, abbreviations, and Montreal Neurological Institute (MNI) coordinates (mm) of the default mode network region of interests (ROIs). Spherical ROIs were generated with a radius of 4 mm centered at the coordinates listed above. ROIs are based on Andrews-Hannah DMN atlas (Andrews-Hanna et al., 2010).

### 2.10. Single-voxel MR spectroscopy

Water-scaled spectroscopy data were evaluated using LCModel Version 6.3 (Provencher, 1993). The absolute metabolite concentrations of guanidinoacetate (Gua), total *n*-acetylaspartate (tNAA), total choline (tCho), total creatine (tCr), and myo-inositol (Ins) were calculated. Voxels were acquired from lesion-free centrum semiovale white matter (NAWM), 38 voxels were from the left hemisphere and 13 from the right one (depending on lesion load). The volume of the voxel was kept constant near 3.375cm^3^ (mean ± SD: 3.36 ± 0.089), but the shape varied patient to patient to fit lesion free areas. Cramer-Rao Lower Bound of 20% was set as exclusion criteria for poor quality spectra.

### 2.11. Statistical analysis

miRNA concentration data from QuantaSoft software were exported and analyzed for quantitative differences between the study groups with IBM SPSS Statistics 21.0. Serum miRNA concentrations in the different patient sub-groups were compared with the Kruskal–Wallis non-parametric test followed by Dunn’s *post hoc* comparison. Voxel-wise statistics of TBSS and ALFF evaluations were performed using a permutation-based non-parametric analysis (randomize). All other statistical analyses were performed using SPSS (IBM Corp., Version 25.0. Armonk, NY). For volumetric and lesion volume analyses, multiple linear regression models were employed with the segmented brain structures or lesion volumes as dependent variable and miRNA amount, age, gender, estimated total intracranial volume (for cerebral WM model), and current therapy as independent variables. Similar statistical approach was used to assess the relationship between brain metabolite concentrations in NAWM and the examined miRNA, using the metabolite concentrations as dependent variable, while miRNA amount, age, gender, disease type, and current therapy as independent variables. For functional connectivity multiple linear regression models were also utilized, using the *z*-standardized correlation strength as dependent variable, while miRNA amount, age, gender, disease type and current therapy as independent variables. The assumptions of multiple linear regression were satisfied, as judged by testing for linearity, independence of errors, outliers, normality assumptions of the residuals, homoscedasticity and multi-collinearity. Significance level was set at *p* < 0.05.

## 3. Results

### 3.1. Serum microRNA levels and their correlation with the clinical outcomes

**Figure 1:**
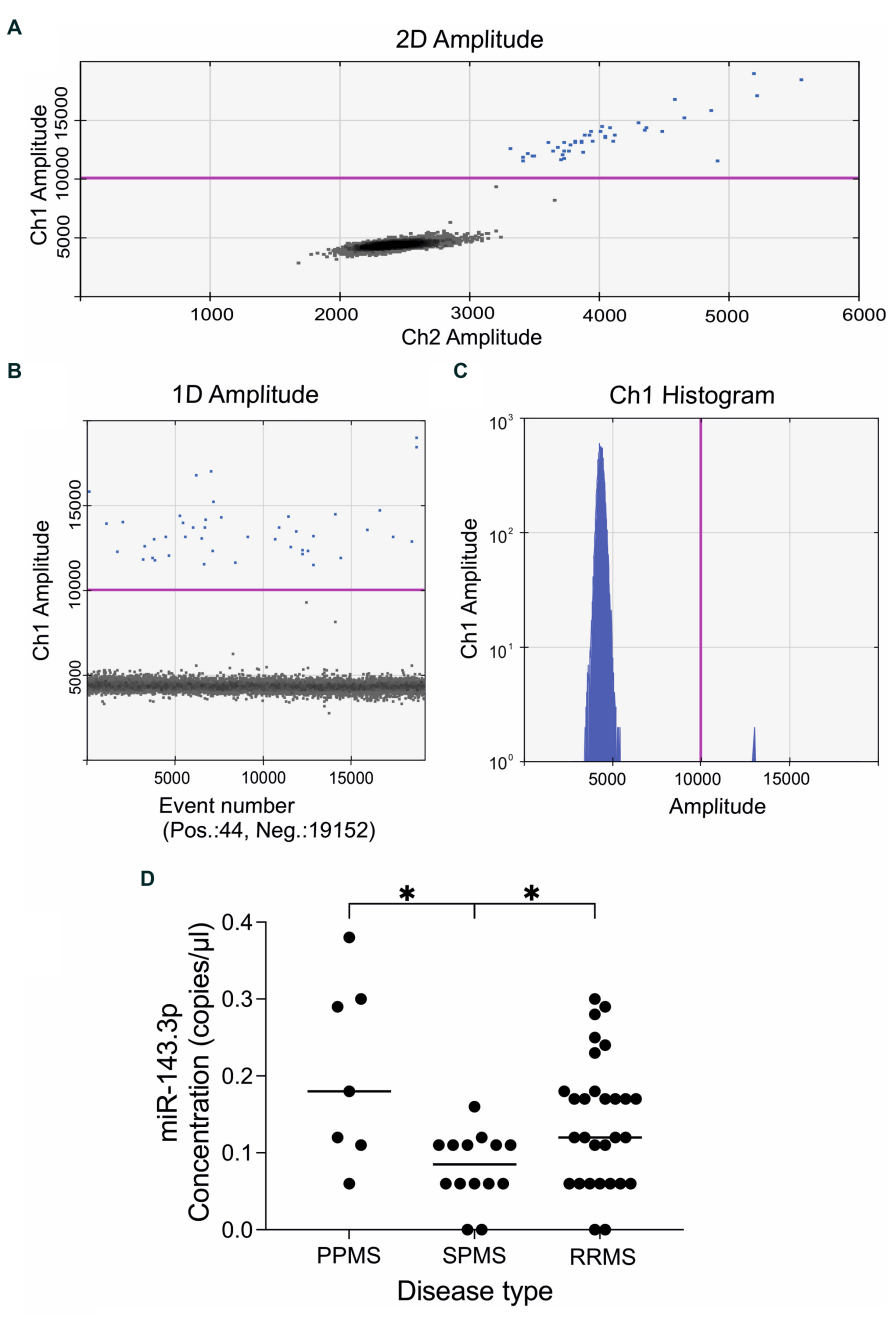
Representative results of the quantitative droplet digital PCR analysis. The plots and graphs were generated by the QuantaSoft analysis software and depict the fluorescent readouts. **(A)** The horizontal and vertical axes represent the fluorescent amplitude in the FAM/EvaGreen and HEX/VIC channels. **(B)** Event numbers are shown on the horizontal axis and fluorescent amplitude is shown on the vertical axis. **(C)** Each axis represents the amplitude in the FAM/EvaGreen channel as a histogram. Data is shown here on a logarithmic scale (vertical axis) for better visualization of the low concentrations of the target miRNA. The threshold line in pink color was set manually at value 10,000 amplitude. Positive droplets are colored with blue, while negative droplets are in black. **(D)** Expression levels of miR143.3p could differentiate between the three main MS subtypes. Circulating miR143.3p levels were significantly lower in the secondary-progressive MS patients compared to the primary-progressive MS (*p* = 0.014) and to relapsing–remitting MS patients (*p* = 0.046). Statistics: Kruskal–Wallis nonparametric test followed by Dunn’s post-hoc analysis. The vertical axis displays the serum levels of miR-143.3p in copies/μl. The disease types are shown in the vertical axis: PPMS, primary-progressive multiple sclerosis; RRMS, relapsing–remitting multiple sclerosis; SPMS, secondary-progressive multiple sclerosis. *p < 0.05.

We measured the expression levels of ten pre-selected miRNAs using droplet digital PCR analysis (Table 1). Representative examples of the ddPCR analysis are displayed on Figure 1. Out of these ten investigated miRNAs, six had either zero serum levels in most patients, or showed very low expression levels (miR-21.3p, miR-146a, miR-181c.3p, miR-181c.5p, miR-375, and miR-629.5p, see Table 1). Therefore, these six miRNAs were excluded from further statistical analyses. Instead, we focused on the remaining four miRNAs, which were detectable at least in 15 individuals, and the comparisons with the MRI findings were tested using these four miRNAs: miR-92a.3p, miR-142.5p, miR-143.3p, and miR-486.5p (Table 1).

First, we examined whether any of these miRNAs could differentiate between the main disease subtypes, i.e., primary-progressive MS (PPMS, *n* = 7), secondary-progressive MS (SPMS, *n* = 14) and relapsing–remitting MS (RRMS, *n* = 29). We found that circulating miR-143.3p levels were significantly lower in the SPMS group compared to the other two groups. Kruskal–Wallis nonparametric test revealed significant difference (*H* = 9.514, *p* = 0.008) between the three main MS subtypes and Dunn’s post-hoc test detected significant differences between the PPMS and SPMS (adjusted *p-*value 0.014) and between the SPMS and RRMS (adjusted *p*-value 0.046) subgroups (Figure 1D).

We also performed correlation analyses between the serum concentrations of miR-92a.3p, miR-142.5p, miR-143.3p, miR-486.5p and the clinical data, i.e., age, age of onset, Expanded Disability Status Scale (EDSS) score, disease duration, and the currently applied disease modifying therapies. In most cases, we could not detect any correlations (Table 4), except, that the expression level of miR-142.5p showed negative correlation (*r* = −0.302, *p* = 0.033) with disease duration and miR-143.3p showed positive correlation (*r* = 0.327, *p* = 0.021) with the EDSS scores (Table 4).

**Table 4:** Correlating serum miRNA levels with clinical parameters.

		Age	Age of onset	Disease duration	Expanded disability status scale	Disease modifying treatment
miR-92a.3p	Pearson Correlation	−0.186	−0.051	−0.185	0.172	0.227
	Significance	0.196	0.723	0.199	0.232	0.113
miR-142.5p	Pearson Correlation	−0.110	0.134	−0.302^*^	0.198	0.177
	Significance	0.447	0.352	0.033^*^	0.168	0.220
miR-143.3p	Pearson Correlation	0.133	0.222	−0.083	0.327^*^	−0.104
	Significance	0.356	0.122	0.564	0.021^*^	0.473
miR-486.5p	Pearson Correlation	0.048	0.236	−0.212	0.277	0.194
	Significance	0.741	0.099	0.139	0.052	0.176

*Correlation is significant at the 0.05 level (two-tailed).

### 3.2. Association of circulating miRNA levels with structural MRI data and cerebral metabolites

**Figure 2:**
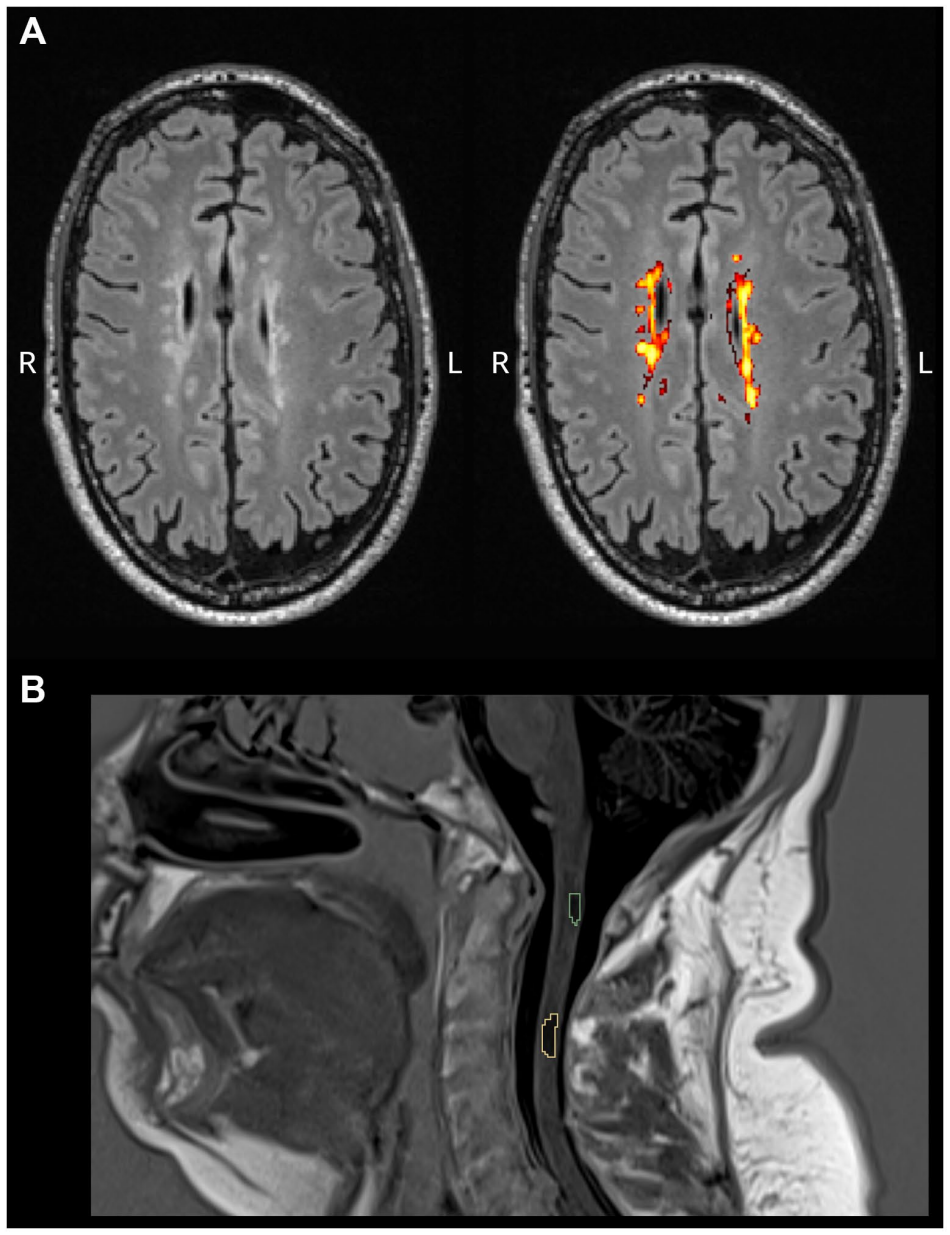
Representative examples of lesion segmentations. Illustrations are presented on FLAIR images for automatic cerebral white matter lesion segmentation **(A)** and using PSIR images for manual cervical spine lesion delineation **(B)**.

First, we examined the relationship between miRNA levels and lesion volumes segmented in the cervical spine and white matter (Figure 2). In the cervical spine, the median of the segmented lesion volume was 300.407 mm^3^ (88–652), and 29 patients had multiple lesions. We found significant associations between miR-92a.3p and miR-486.5p levels and total lesion volumes within the cervical spine (*t* = 2.040, *p* = 0.05 and *t* = 2.384, *p* = 0.023, respectively). Higher miRNA levels were associated with greater lesion volumes. Neither miR-142.5p, nor miR-143.3p showed any significant association with the total cervical lesion volume. We did not find any association between the examined miRNA levels and the LST segmented total lesion volumes.

**Figure 3:**
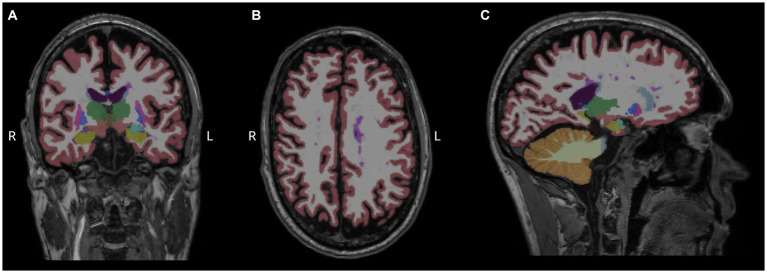
Measurement of regional brain volumes. Results of Freesurfer-based automatic segmentation shown as an overlay in a representative patient. Coronal **(A)**, axial **(B)**, and sagittal **(C)** slices are shown using aseg.mgz as overlay with default Freesurfer lookup table. Magenta color represents white matter hypointensities, predominently visible in the centrum semiovale, close to the left lateral ventricle.

Then, we performed a linear regression analysis between miRNA levels and regional brain volumes. Forty reliably segmented structures were extracted from aseg.mgz, omitting the brainstem, but none of the examined miRNA levels had significant association with the segmented volumes (Figure 3).

Next, we conducted a nonparametric permutation based statistical analysis to assess whether miRNA levels show any significant association with microstructural parameters of the white matter, which have been investigated with diffusion tensor imaging. In the statistical analysis, gender, age, total cerebral lesion volume, and current therapy were included as nuisance variables. None of the examined miRNA showed any significant association with the microstructure of the white matter skeleton.

Finally, we examined the relationship between circulating miRNA levels and cerebral metabolites which have been measured with single-voxel MR spectroscopy. We found a single significant association between cerebral metabolite concentrations and circulating miRNAs, namely reduced miR-142.5p levels correlated with decreased total creatinine concentrations (*t* = 2.275, *p* = 0.029).

### 3.3. Associations between miRNA levels and functional connectivity

**Figure 4:**
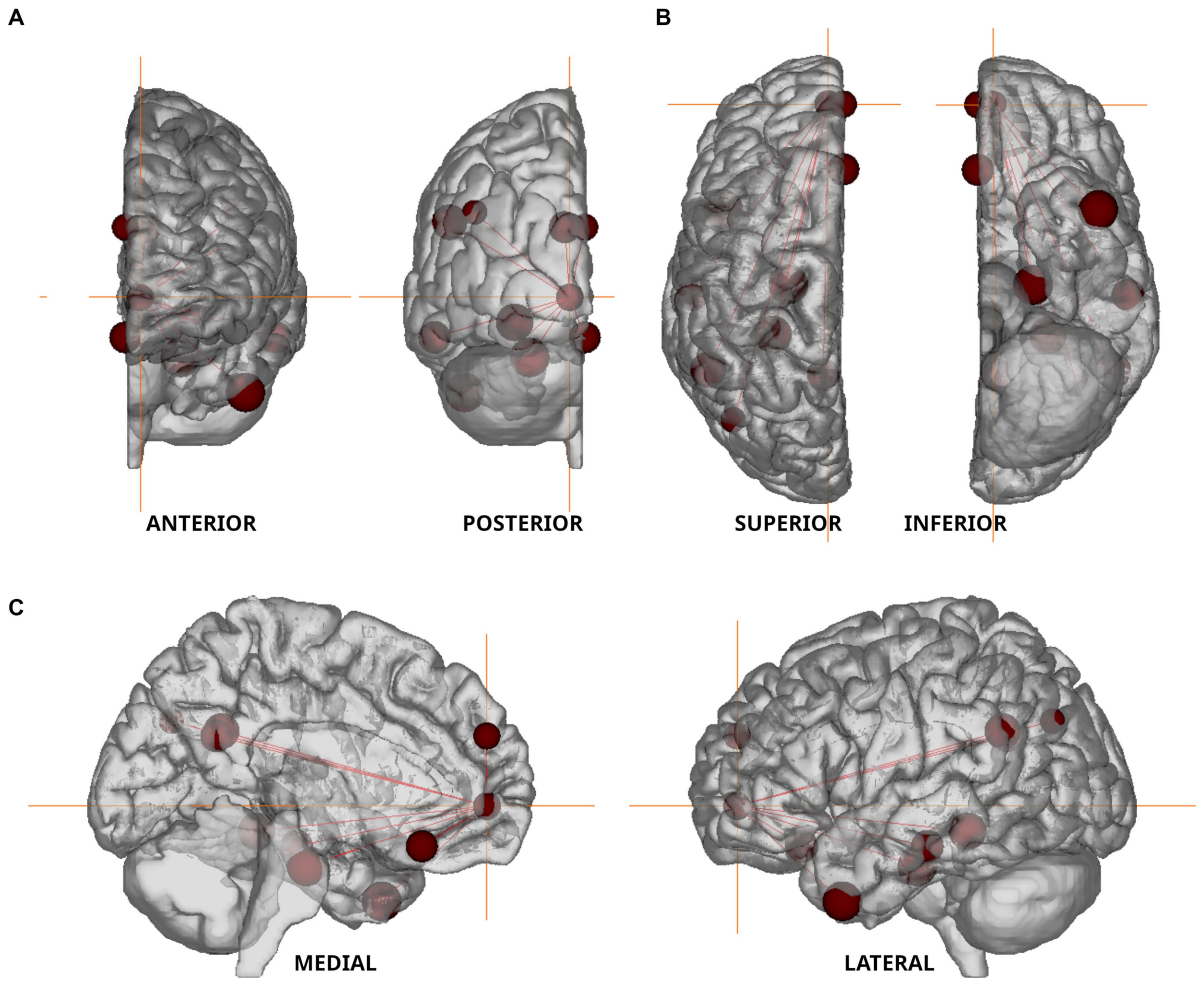
Nodes of the Default Mode Network used in resting state fMRI analysis. The nodes defined by Andrews-Hanna et al. (2010) are shown in anterior–posterior **(A)**, superior–inferior **(B)**, and medio-lateral **(C)** views. Orange colored crosshair are positioned on the anterior medial prefrontal cortex.

Permutation-based non-parametric tests found no significant association between voxelvise ALFF values and miRNA concentrations. However, functional connectivity strengths between the 11 nodes of the default mode network (Figure 4 and Table 3) showed significant associations with three out of the four examined miRNAs. All models were adjusted for age, gender, current therapy and disease type.

We found a negative association between miR-92a.3p and miR-486.5p levels and functional connectivity strength measured between the lateral temporal cortex and posterior inferior parietal lobule (*t* = −2.232, *p* = 0.032 and *t* = −2.31, *p* = 0.027, respectively). In contrast, serum miR-142.5p concentrations showed a positive association with functional connectivity strength measured between the retrosplenial cortex and temporal pole (*t* = 2.316, *p* = 0.027). MiR-143.5p had no significant association with functional connectivity strengths within the default mode network.

## 4. Discussion

Here, we report significant associations between circulating levels of three serum miRNA and specific MRI outcomes in MS patients. Specifically, we found that (1) miR-92a.3p correlated with total white matter lesion volume within the cervical spine, and with functional connectivity strength between the lateral temporal cortex and posterior inferior parietal lobule; (2) miR-142.5p was associated with total creatinine concentrations and with functional connectivity strength between the retrosplenial cortex and temporal pole; (3) miR-486.5p levels were associated with total white matter lesion volumes within the cervical spine and with functional connectivity strength between the lateral temporal cortex and posterior inferior parietal lobule. Furthermore, we found that circulating miR-143.3p levels could differentiate between the three main MS subtypes, i.e., serum miR-143.3p levels were the lowest in the secondary-progressive MS subgroup. We could also detect a positive correlation between miR-143.3p levels and the EDSS scores of the patients whereas, miR-142.5p levels showed a negative correlation with disease duration.

The principal aim of our present study was to replicate the earlier findings of Regev and co-workers, who reported significant associations between miRNA levels and structural MRI alterations in MS patients (Regev et al., 2017). Specifically, they found numerous miRNAs to be associated with either lesion volumes or brain atrophy (Table 1), although none of these associations remained significant when they corrected for multiple comparisons. Here, we focused on the miRNAs that showed the strongest associations with the MRI data in the Regev et al. (2017) study, and we expected to replicate their findings. However, in our cohort we could find only a few similarities. For example, they reported that the pathogenic miRNA, miR-92a.3p, showed a positive correlation with lesion severity (T1:T2 ratio). In our case, we also found a significant correlation between miR-92a.3p levels and total white matter lesion volume within the cervical spine. Notably, miR-92a.3p belongs to the miR-17-92 cluster, which is overexpressed in B cells and in the plasma of patients with MS (Sievers et al., 2012; Gandhi et al., 2013). This miRNA plays role by repressing gene targets known to regulate immune cell activity and prevent inflammation (Regev et al., 2016). Furthermore, miR-92a-1 could differentiate between SPMS from RRMS, and it showed a strong association with EDSS scores as well as disease duration (Gandhi et al., 2013). But, in our samples, we could not replicate this finding. Regev et al. (2017) reported a positive correlation with lesion severity also for miR-486.5p, which we could partially replicate as in our case miR-486.5p levels were associated with total white matter lesion volumes within the cervical spine. Previously, it has been reported that miR-486.5p was upregulated in MS patients (Regev et al., 2016, 2018) and increased expression of miR-486.5p can downregulate phosphatase and tensin homologue (PTEN) and forkhead Box O1 (FOXO1) which are important participants in molecular pathways and their impaired functioning can cause pathogenic changes such as T cell dysfunction (Alexander et al., 2014; Wang et al., 2015). Notably, in our previous study examining the same cohort, we found correlation between brain volumes and inflammatory marker levels in the cerebrospinal fluid (Orsi et al., 2021b), which data suggests that miRNAs regulating inflammatory responses may show stronger correlation with the MRI findings.

The most important novelty of our study is that three miRNAs were associated with functional connectivity strengths of specific nodes within the default mode network. It is well documented that resting state functional connectivity alterations correlate with the clinical symptoms in MS patients (e.g., Tommasin et al., 2020; Jandric et al., 2022; Romanello et al., 2022), but to our best of knowledge our present finding is the first in the literature to report on associations between serum miRNAs and functional connectivity in MS patients. There are only a handful of studies in the literature documenting associations between these two types of neuroimaging-epigenetic biomarkers. In a recent study, He et al. (2021) investigated patients with depression who suffered childhood maltreatment, a key risk factor to develop depression, and they found that the severity of childhood maltreatment was linked with serum miR-9 levels, and also to intrinsic amygdala functional connectivity. Another study found correlations between peripheral levels of miR-1202 and functional connectivity of specific cortical regions in the context of depression and antidepressant response (Lopez et al., 2017). There are also reports that homozygous carriers of a specific gene variant, the MIR137 risk allele (TT), which confers a high risk for schizophrenia, display significantly different cortico-limbic functional connectivity compared with heterozygous individuals (Liu et al., 2014; Mothersill et al., 2014). Notably, MIR137 is one of a group of genes that encode miRNAs. Clearly, further studies are needed to establish the significance of these findings and their relevance in MS.

We also found an association between miR-142.5p levels and total brain creatinine concentrations as measured by single-voxel MR spectroscopy. To our best of knowledge there is no comparable finding in the literature and future studies are necessary to validate this finding. What is known, is that miR-142.5p has been associated with Hashimoto’s thyroiditis (Zhu et al., 2016; Bayomy et al., 2022), the most common type of autoimmune disease, which is typically associated with subsequent hypothyroidism and thyroid function is known to influence creatinine metabolism (Hall et al., 1996; Verhelst et al., 1997). The co-occurrence of autoimmune thyroid diseases is not uncommon in MS patients (Niederwieser et al., 2003; Sloka et al., 2005).

We also found that miR-143.3p levels were significantly lower in the SPMS patients compared to the PPMS and RRMS subgroup. A somewhat comparable finding has been reported recently by Muñoz-San Martín et al. (2022), who found that miR-143.3p was significantly downregulated in the cerebrospinal fluid of primary-progressive MS patients compared to other neurologic disease, but secondary-progressive MS patients were not included in their cohort. Several studies pointed out miR-143.3p as a potential biomarker for various neurological disorders such as Alzheimer’s disease (Cheng et al., 2015; Lusardi et al., 2017), acute ischemic stroke (Tiedt et al., 2017), amyotrophic lateral sclerosis (Waller et al., 2017), or cerebral atherosclerosis (Gao et al., 2019), and also for immune-mediated inflammatory disorders (Martínez-Hernández et al., 2020).

The four miRNAs which we found to correlate with the MRI measures have already been identified by several previous MS studies (Table 1). For example, miR-92a-1 showed an association with EDSS scores and disease duration and different expression was found in RRMS patients versus healthy controls and SPMS patients (Gandhi et al., 2013). Altered expression of miR-142.5p in MS patients have been repeatedly demonstrated. It was upregulated in active MS lesions (Junker et al., 2009) and in the frontal white matter of MS patients (Talebi et al., 2017). Reduced CSF levels of miR-142.5p were associated with higher number of T2 lesions whereas, increased CSF levels correlated with higher number of gadolinium+ active lesions (Muñoz-San Martín et al., 2022). Serum levels of miR-142.5p were upregulated in RRMS samples compared with other neurological disorders (Muñoz-San Martín et al., 2022). Regev and co-workers reported a significant difference of serum miR-142.5p levels between SPMS patients and healthy individuals and that it correlates well with EDSS scores (Regev et al., 2018). A recent study, found that miR-143.3p was downregulated in the CSF of PPMS patients compared with other neurological disorders (Muñoz-San Martín et al., 2022). In case of miR-486.5p, it was upregulated in MS compared to healthy controls and to other neurologic diseases, as well as compared to other inflammatory diseases and it correlated with the EDSS scores (Regev et al., 2016, 2018). These accumulating data highlight the potential diagnostic value of these miRNAs in MS.

The present study has several limitations which should be kept in mind, when we interpret the results. Major limitations were that this was a single-center study with relatively low sample size and that we examined only 10 pre-selected miRNAs. Further limitation is the lack of control group in our study. Our major difficulty was that six out of the 10 preselected miRNAs were not detectable in most patients (Table 1). Therefore, we had to exclude these six miRNAs from the statistical comparisons with the MRI measurements. The reason for this issue with low detectability likely to have both methodical and biological causes. Many miRNAs, which are synthetized in the CNS, are likely to cross the blood–brain barrier, but then, since the blood volume is much greater than the CSF volume, these miRNAs will have a much more diluted concentration in serum than in the CSF (Stoicea et al., 2016). Additional methodical problem is that different laboratories often use different reagents and instruments to extract the appropriate quality of miRNAs from biological fluids, which may also influence the detection levels of miRNAs (Bryzgunova et al., 2021; Ban and Song, 2022). Lifestyle factors, such as diet, stress, physical activity, smoking and alcohol consumption may also affect the expression levels of miRNAs (Panico et al., 2021).

In summary, we could only partially replicate the earlier findings of the Regev et al. (2017) study, who reported correlations between the serum levels of all these 10 miRNA and structural CNS alterations (lesion volume and brain atrophy). A major novelty of our present study is that we report here associations between specific circulating miRNA levels and functional MRI finings (functional connectivity within the default mode network and a brain metabolite concentration). Further studies are needed to validate our present data.

## Data availability statement

The raw data supporting the conclusions of this article will be made available by the corresponding author upon reasonable request.

## Ethics statement

The studies involving humans were approved by the local Research Ethics Committee of the University of Pécs. The studies were conducted in accordance with the local legislation and institutional requirements. The participants provided their written informed consent to participate in this study.

## Author contributions

LG: investigation, data curation, formal analysis, methodology, visualization, and writing – original draft. GO: investigation, data curation, formal analysis, methodology, project administration, visualization, and writing – original draft. TC: investigation, resources, data curation. KG: methodology, resources, supervision, and writing – review and editing. ZI: conceptualization, funding acquisition, project administration, resources, supervision, writing – review and editing. BC: conceptualization, funding acquisition, project administration, supervision, writing – original draft, and writing – review and editing. All authors contributed to the article and approved the submitted version.

## Footnotes

^1^ www.statistical-modelling.de/lst.html

^2^ http://rfmri.org/dpabi

## Abbreviations

ALFF: amplitude of low-frequency fluctuation
BET: Brain Extraction Tool
cDNA: complementary DNA
CNS: central nervous system
CSF: cerebrospinal fluid
CUDA: Compute Unified Device Architecture
DALYs: disability-adjusted life years
DARTEL: diffeomorphic anatomical registration through exponentiated lie
ddPCR: Droplet digital PCR
DMN: default mode network
DMT: disease modifying therapy
DPARSF: Data Processing Assistant for Resting-State fMRI
DTI: diffusion tensor imaging
EDSS: Expanded Disability Status Scale
FA: fractional anisotropy
FLAIR: fluid-attenuated inversion recovery
fMRI: resting-state functional MRI
FOV: field of view
FSL: FMRIB Software Library
FWHM: full width at half maximum
1H-MRS: proton magnetic resonance spectroscopy
Ins: myo-inositol
IQR: interquartile range
LNA: locked nucleic acid
LST: Lesion segmentation tool for SPM
MD: mean diffusivity
miRNA: microRNA
MNI: Montreal Neurological Institute
MPRAGE: magnetization-prepared rapid acquisition with gradient echo
MRI: magnetic resonance imaging
MRS: single voxel point resolved spectroscopy
MS: multiple sclerosis
NAWM: normal-appearing white matter
NPC: no primer control
NTC: no template control
PCR: polymerase chain reaction
PPMS: primary-progressive multiple sclerosis
PSIR: phase-sensitive inversion recovery
RD: radial diffusivity
ROI: regions of interest
RRMS: relapsing–remitting multiple sclerosis
rs-fMRI: resting-state functional MRI
SD: standard deviation
SPMS: secondary-progressive multiple sclerosis
tCho: total choline
tCr: total creatine
tNAA: total *n*-acetylaspartate
TBSS: tract-based spatial statistics
TOPUP: Topup toolbox of the FMRIB Software Library
WM: white matter
